# CRISPR-Cas9 DNA Base-Editing and Prime-Editing

**DOI:** 10.3390/ijms21176240

**Published:** 2020-08-28

**Authors:** Ariel Kantor, Michelle E. McClements, Robert E. MacLaren

**Affiliations:** 1 Nuffield Laboratory of Ophthalmology, Nuffield Department of Clinical Neurosciences & NIHR Oxford Biomedical Research Centre, University of Oxford, Oxford OX3 9DU, UK; michelle.mcclements@eye.ox.ac.uk (M.E.M.); robert.maclaren@eye.ox.ac.uk (R.E.M.); 2 Oxford Eye Hospital, Oxford University Hospitals NHS Foundation Trust, Oxford OX3 9DU, UK

**Keywords:** CRISPR/Cas9, base-editing, prime-editing, adeno-associated vector, genome engineering, gene therapy

## Abstract

Many genetic diseases and undesirable traits are due to base-pair alterations in genomic DNA. Base-editing, the newest evolution of clustered regularly interspaced short palindromic repeats (CRISPR)-Cas-based technologies, can directly install point-mutations in cellular DNA without inducing a double-strand DNA break (DSB). Two classes of DNA base-editors have been described thus far, cytosine base-editors (CBEs) and adenine base-editors (ABEs). Recently, prime-editing (PE) has further expanded the CRISPR-base-edit toolkit to all twelve possible transition and transversion mutations, as well as small insertion or deletion mutations. Safe and efficient delivery of editing systems to target cells is one of the most paramount and challenging components for the therapeutic success of BEs. Due to its broad tropism, well-studied serotypes, and reduced immunogenicity, adeno-associated vector (AAV) has emerged as the leading platform for viral delivery of genome editing agents, including DNA-base-editors. In this review, we describe the development of various base-editors, assess their technical advantages and limitations, and discuss their therapeutic potential to treat debilitating human diseases.

## 1. Introduction

The recent discovery of the clustered regularly interspaced short palindromic repeats (CRISPR)-Cas system has revolutionized the field of molecular biology and medicine [1]. CRISPR-mediated genome editing involves the generation of a Cas9-induced double-strand break that is repaired by non-homologous end joining (NHEJ) mechanisms or by homology directed repair (HDR) [2,3,4]. Although HDR can be harnessed to insert a specific DNA template for precise restoration of the DNA sequence, this pathway is characterized by limited efficiency and high rates of undesired insertion or deletion (indel) mutations that nullify the potential benefit from repairing the mutation [5,6]. Moreover, due to reliance on homologous recombination, HDR-mediated editing is restricted to dividing cell types, limiting the range of diseases that can be targeted [7]. Recently, CRISPR/Cas-mediated single-base-pair editing systems have been devised to bypass these limitations [8,9]. Two classes of DNA base-editors (BEs) have been described thus far: cytosine base-editors (CBEs) and adenine base-editors (ABEs). These BEs can install all four transition mutations. Prime-editors (PEs) are the latest addition to the CRISPR genome-engineering toolkit and represents a novel approach to expand the scope of donor-free precise DNA editing to not only all transition and transversion mutations, but small insertion and deletion mutations as well [10]. Collectively, DNA base-editing and prime-editing tools enable precise nucleotide substitutions in a programmable manner, without requiring a donor template.

DNA base-editing and prime-editing has remarkable potential as a therapeutic tool to correct disease-causing mutations in the human genome. Over 25% of human pathogenic SNPs can be corrected by targeting the four transition mutations, and in principle prime-editing could correct up to 89% of known genetic variants associated with human disease [9,10]. DNA base-editing and prime-editing may prove to be particularly well adapted for correction of large genes, where the vector-mediated delivery of the target gene is not feasible due to the limited packaging capacity of viral vectors [11]. Moreover, base-editing can be applied to autosomal dominant diseases, where gene augmentation is not a suitable approach due to the requirement for silencing or ablating the defective gene in autosomal dominant diseases. In addition, recently developed base-editor adaptations such as “CRISPR-Pass” may prove advantageous in the correction of diseases characterized by premature terminations or alternative splicing [12]. Indeed, the therapeutic potential of DNA base-editors and prime-editors is tremendous [8,9,13].

Development of safe and efficient delivery systems is crucial for the success of CRISPR-Cas base-editing and prime-editing in the clinic [14,15]. The use of viruses to deliver DNA encoding base-editors is a promising delivery modality and builds off the successes of gene therapy approaches. Due to its broad tropism, well-studied serotypes, and limited immunogenicity, AAV has emerged as the leading platform for viral delivery of genome editing agents [16]. Here, we review the CRISPR/Cas base-editing toolkit, describe the adeno-associated vector as a delivery vehicle for BE-mediated gene therapy, and discuss their therapeutic potential to treat inherited human diseases.

## 2. CRISPR Genome Editing

Discovery of the mechanisms of the clustered regularly interspaced short palindromic repeats (CRISPR)-Cas9 system in the bacterial immune system, and subsequent adaptation into a powerful gene editing tool has revolutionized the field of molecular biology and generated excitement for the potential of novel therapeutic approaches to treat human conditions [17,18,19]. The CRISPR/Cas system encompasses a variety of components which differ widely in mechanisms of action and offer therapeutic potential by direct genome interaction and/or editing. Despite the complexity of the Cas family, all systems share a requirement for CRISPR RNA (crRNA) for defined target specificity whilst type II variants have an additional requirement for trans-activating RNA (tracrRNA), which forms a scaffold structure [20]. For gene editing applications, the two CRISPR RNAs described above are joined as one small guide RNA (sgRNA), which greatly simplifies delivery. The Cas9: sgRNA complex randomly interrogates DNA in the cell, searching first for the appropriate protospacer adjacent motif (PAM), a short motif adjacent to the target sequence. Upon recognition of the PAM sequence, the Cas9 protein unwinds the DNA, allowing the Cas9-associated sgRNA to hybridize with the exposed DNA strand (the protospacer). If the DNA sequence matches the sgRNA target sequence, the HNH and RuvC catalytic domains of the endonuclease cleave both strands of the target DNA, generating a double strand break [18]. This break is then repaired by the host cell by NHEJ or HDR mechanisms, as discussed later.

One constraint of Cas9 is its dependency on the aforementioned PAM sequence to bind DNA. Because of the simple 5′-NGG-3′ PAM sequence requirements, S. pyogenes’ Cas9 (SpCas9) is used in many different applications. However, scientists are actively exploring other CRISPR systems to identify Cas9-like effector proteins that may have differences in their sizes, PAM requirements, and targeting specificity. Naturally found Cas9 variants are large proteins, which adds particular limitation when it comes to packaging and delivery to target cells. For example, the SpCas9 protein is 4098 base-pairs (bps), making therapeutic delivery challenging due to the limited packaging capacity of adeno-associated vector (reviewed below). To this end, the discovery of smaller variants such as 3246 bp Cas9 from *Staphylococcus aureus* (SaCas9) and 2952 bp Cas9 from *Campylobacter jejuni* (CjCas9) provides great therapeutic potential [21,22,23]. However, these smaller Cas9 proteins require more complex PAM sequences, limiting targeting scope compared to SpCas9 despite the size reduction. The SaCas9 requires a 5′-NNGRRT-3′ PAM sequence whereas CjCas9 requires a 5′-NNNNACAC-3′ PAM sequence. Moreover, in a direct comparison of SpCas9, SaCas9, and CjCas9 orthologues in human embryonic kidney cells carrying a reporter of eGFP (HEK293-eGFP), our group recently found that SpCas9 demonstrates the highest efficacy among the tested Cas9 variants [24].

While exploration of natural Cas diversity provides one avenue for expanding and improving PAM coverage, the efficiency of Cas varies, and to date, no variants have surpassed SpCas9 efficacy. Thus, complimentary evolution-based and structure-guided engineering approaches have been employed to modify and improve Cas9 effector scope [25,26,27]. For example, Kleinstiver et al. evolved three SpCas9 variants (VQR, EQR and VRER) which recognize the novel PAM sequences NGAN/NGNG, NGAG and NGCG, respectively [27]. These evolved SpCas9 variants, together with recently reported variants, in principle enable the targeting of most NR PAM sequences [28,29,30] Recently, Walton et al. reported the evolution of a two novel SpCas9 variants: SpG, capable of targeting an expanded set of NGN PAMs, and a nearly-PAMless variant called SpRY [31]. Importantly, SpRY allowed for correction of a wide range of pathogenic mutations located in previously “un-targetable” regions of the genome.

In addition to expanding the targeting scope of CRISPR tools, researchers are actively developing novel ways to increase the targeting specificity and minimize the off-target effects of the CRISPR-Cas9 system. Several studies have described Cas9 variants evolved to increase the targeting specificity [32,33]. In addition to engineering approaches of the Cas9 protein, the sgRNA scaffold can also be modified to increase the targeting specificity [34]. For example, the secondary structure of the gRNA spacer region can be modified to increase the thermodynamic barrier to gRNA binding at off-target sites [35]. Furthermore, increasing or decreasing the length of the sgRNA guiding sequence by a few base pairs can enhance the targeting specificity [36,37]. Finally, modifying the delivery platform of the CRISPR-Cas9 construct can further enhance targeting. For example, delivery of the Cas9-sgRNA complex as a ribonucleotide protein (RNP) complex results in more transient activity of Cas9 and lower rates of off-target cleavage [38,39].

As described above, genome editing mediated by CRISPR/Cas9 requires targeted induction of a DNA double strand break. In Eukaryotic organisms, Cas9-induced DSBs are repaired either by error prone non-homologous end joining or homology-directed repair [2,4]. Typically, CRISPR-induced DSBs are repaired by NHEJ, an efficient and prevalent mechanism in human cells that results in random insertions or deletions and gene disruption in the target region. HDR is a well-established mechanism that can be harnessed to insert a specific DNA template for precise restoration of the DNA sequence, with limited off-target activity or on-target alterations [5]. HDR-mediated insertion, however, requires the presence of the correct template and is typically characterized by lower efficiency than NHEJ repair. In addition, the correction of a point-mutation by HDR has shown to be highly ineffective particularly in nondividing cells, and the dsDNA break formation by Cas9 nuclease generates undesired indel mutations at a substantial frequency that annuls the potential benefit from corrected mutation [40,41]. DNA base-editing and prime-editing enable a targeted tool to bypass these challenges.

## 3. DNA Base-Editing

DNA base-editors encompass two key components: a Cas enzyme for programmable DNA binding and a single-stranded DNA modifying enzyme for targeted nucleotide alteration. Two classes of DNA base-editors have been described: cytosine base-editors and adenine base-editors. Collectively, all four transition mutations (C→T, T→C, A→G, and G→A) can be installed with the available CRISPR/Cas BEs (Table 1). Recently, Kurt et al. describe the engineering of two novel base-editor architectures that can efficiently induce targeted C-to-G base transversions [42]. In addition, recent studies report dual base-editor systems for combinatorial editing in human cells [43,44,45]. Together, these new base-editors expand the range of DNA base-editors to transversion mutations and may allow for targeting of more complex compound edits than are currently achievable by a single DNA base-editor.

### 3.1. Cytosine Base-Editors

**Figure 1:**
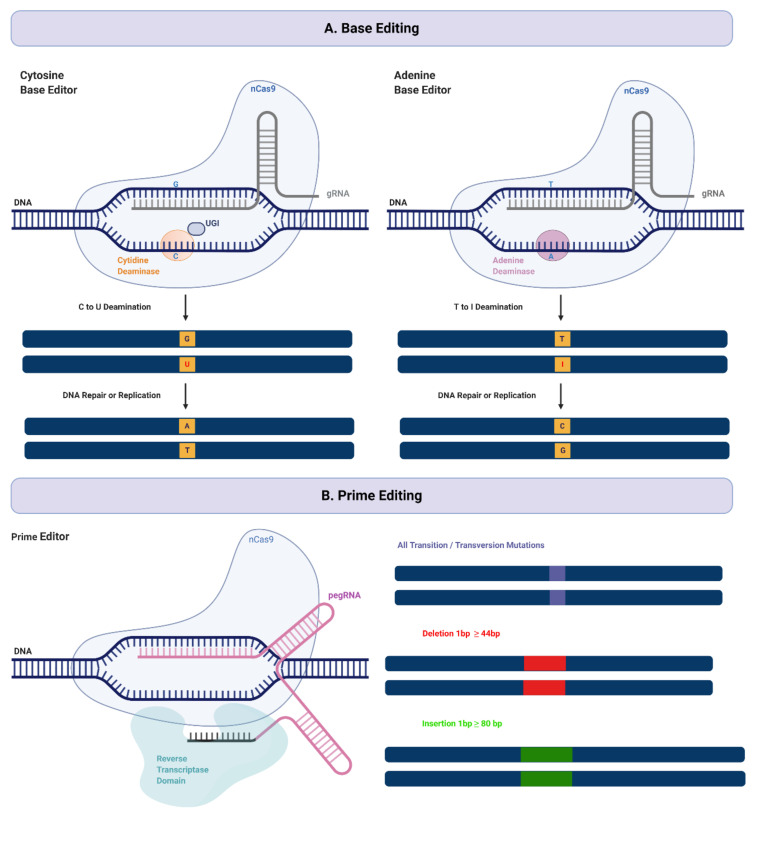
CRISPR DNA Base-Editing Tools. (**A**) DNA Base-editing. DNA base-editors encompass two key components: a Cas enzyme for programmable DNA binding and a single-stranded DNA modifying enzyme for targeted nucleotide alteration. Two classes of DNA base-editors have been described: cytosine base-editors and adenine base-editors. Cytosine deamination generates uracil, which base pairs as thymidine in DNA. Fusion of uracil DNA glycosylase inhibitor (UGI) inhibits the activity of uracil N-glycosylate (UNG), thus increasing the editing efficiency of cytosine base-editing in human cells. Adenosine deamination generates inosine, which has the same base pairing preferences as a guanosine in DNA. Collectively, cytosine and adenine base-editing can install all four transition mutations (C→T, T→C, A→G, and G→A). (**B**) Prime-editing. Prime-editors use an engineered reverse transcriptase fused to Cas9 nickase and a prime-editing guide RNA (pegRNA). The pegRNA contains the sequence complimentary to the target sites that directs nCas9 to its target sequence as well as an additional sequence spelling the desired sequence changes. Prime-editors expand the scope of DNA editing to not all transition and transversion mutations, as well as small insertion and deletion mutations.

The first-generation base-editor (CBE1) was developed by Liu and co-workers in 2016 [9,46]. It was engineered by fusing a rat-derived cytosine deaminase Apolipoprotein B MRNA Editing Enzyme Catalytic Subunit 1 (APOBEC1) to the amino terminus of catalytically deficient, or “dead”, Cas9 (dCas9) (Figure 1). In a narrow window of the non-targeted strand, CBE1 deaminates cytosine to uracil. Uracil is then recognized by cell replication machinery as a thymine, resulting in a C-G to T-A transition. Importantly, although CBE1 mediates efficient, targeted base-editing in vitro (up to 37% editing with a 1.1% indel formation rate), it is not effective in human cells [46]. This decrease is largely due to cellular-mediated repair of the U-G intermediate in DNA by the base excision repair (BER) pathway. BER of U-G in DNA is initiated by uracil N-glycosylate (UNG), which recognizes the U-G mismatch and cleaves the glycosidic bond between the uracil and the deoxyribose backbone of DNA, resulting in reversion of the U-G intermediate created by the base-editor back to the C-G base pair [78,79]. Keeping in view the low editing efficiency and limitations of CBE1, a series of improved base-editors were developed further. To improve base-editing efficiency, a second-generation cytosine base-editor (CBE2) was developed by fusing an uracil DNA glycosylase inhibitor (UGI) to the C-terminus of BE1, inhibiting the activity of UDG. The inhibition of BER by BE2 resulted in a threefold increase in editing efficiency in human cells [46] To further improve editing efficacy, BE3 was developed by restoring histidine at position 840 (H840, HNH catalytic domain) in dCas9 to generate a base-editor that uses Cas9 nickase (nCas9). This variant induces a nick in the G-containing strand of the U-G intermediate (non-edited DNA strand) to bias cellular repair of the intermediate towards a U-A outcome, further converted to T-A during DNA replication. This modification further increased editing efficiency by six-fold in BE3 over BE2. The use of nCas9 also exhibited an increase in indel frequency of 1.1% as compared to 0.1% in BE2; however, this is still a low rate that is much less frequent than indels induced by DSBs [46].

Further optimization of CBE was performed to reduce indel formation during base-editing, improve editing efficiency, and narrow the editing window. An improved fourth-generation cytosine base-editor (CBE4) was generated by fusing an additional copy of UGI to the N terminus of nCas9 with an optimized 27 bp linker [63]. YEE-BE3 was developed by screening several mutations previously reported to modulate the catalytic activity of cytosine deaminases in the APOBEC family to generate an improved rAPOBEC1 with a narrower editing window and reduced “bystander editing” compared to CBE3. Gam, a DNA-binding protein from bacteriophage Mu, can form a complex with free-ends of DBSs, thus preventing NHEJ-mediated repair and reducing indel formation [80]. These changes resulted in BE4-Gam, which is characterized by higher base-editing efficiency, increased product purity, and decreased indel frequency [49]. However, Gam binding has been shown to induce the death of DSB-containing cells [81], which may reduce its adaptability towards therapeutic applications.

Separately, Koblan et al. added two nuclear localization signals (NLS) to nCas9 and performed codon-optimization and ancestral sequence reconstruction on APOBEC, yielding BE4max and ancBE4max [72]. Another base-editing system, Target-AID (activation-induced cytidine deaminase), was developed and composed of nCas9, *Petromyzon marinus* cytidine deaminase 1 (pmCDA1), which is similar to rAPOBEC1 in structure and function [8]. The use of alternative cytosine deaminase enzymes yields base-editors with alternative sequence motif preference and the ability to efficiently edit methylated cytosines. Most recently, Liu and colleagues used phase assisted continuous evolution (PACE) to evolve CBEs and generate evoAPOBEC1-BE4max, which can efficiently edit cytosine in G/C sequences (a disfavored context for wild-type APOBEC1 deaminase) and evoFERNY-BE4max, a smaller deaminase that edits efficiently in all tested sequence contests [73] (Table 1).

To increase the number of targetable bases, researchers have developed base-editors incorporating different CRISPR-associated nuclease enzymes. CBEs based on SpCas9 are limited by their G/C-rich PAM sequence. In order to expand the scope of base-editing, Li et al. generated a Cpf1-based cytosine deaminase base-editor by fusing catalytically inactive LbCpf1 (dLbCpf1) or dAsCpf1 with rAPOBEC1 and UGI (creating dLbCpf1-BE0 and dAsCpf1-BE0) [74]. A variety of engineered Cas9 variants with altered PAM sequences and improved cleavage specificity have been developed and may allow for further expansion of the targeting scope of CRISPR-base-editing reagents [28,29,30,82,83,84,85,86]. These constructs enable efficient single-vector AAV delivery and may prove especially useful for therapeutic applications that are constrained by viral-vector packaging capacity (Table 2).

### 3.2. Adenine Base-Editors

The cytosine base-editor is limited to installing a C-G to T-A mutation, greatly restricting the range of correctable disease-causing mutations. Importantly, methylated cytosines are vulnerable to high rates of spontaneous cytosine deamination [87], and nearly half of all pathogenic point mutations in principle can be reversed using an ABE to convert an A-T base pair back into a G-C base pair. As such, base-editing capabilities and study of genetic diseases were further expanded by the development of a new class of adenine base-editors that could induce A to G conversions [13]. ABE-mediated DNA editing operates under a similar mechanism as CBE. The ABE-dCas9 fusion binds to a target DNA sequence in a guide RNA-programmed manner, and the deoxyadenosine deaminase domain catalyzes an adenine to inosine transition. In the context of DNA replication, inosine is interpreted as guanine, and the original A-T base pair may be replaced with a G-C base pair at the target site (Figure 1). Unlike cytosine deaminases, ssDNA adenosine deaminase enzymes do not occur in nature. Attempts at utilizing RNA adenosine deaminases to act on DNA resulted in no detectable RNA editing [13]. David Liu and group overcame this limitation through extensive protein engineering and directed evolution of *Escherichia coli* tRNA adenosine deaminase, TadA (ecTadA). EcTadA converts adenine to inosine in the single-stranded anticodon loop of tRNA^ARG^, and shares sequence similarity with the APOBEC family. The first-generation adenine base-editors were developed through an antibiotic resistance complementation approach in bacteria. To test TadA on a DNA target, *E. coli* cells were equipped with TadA mutants and defective antibiotic resistance genes. To grow in the presence of antibiotic, a mutant TadA-dCa9 fusion had to correct the targeted adenine in a mutant chloramphenicol resistance gene. The first-generation ABE (ABE1.2) was generated by fusing the evolved TadA variant (TadA*) to the N-terminus of nCas9 through XTEN (a 16 amino acid linked used in BE3), with the C terminal of nCas9 fused with a nuclear localization signal (TadA*-XTEN-nCas9-NLS) [13]. In comparison with cytosine base-editing, adenine base-editing by ABE yields a much cleaner product that has virtually no indels, and there are no reports of significant off-target (A-to-non-G) edits to date. Consistent with this observation, unlike UGI-mediated inhibition of UDG in CBEs, ABE editing in cells lacking alkyl adenine DNA glycosylase (AAG), the enzyme known to recognize and remove inosine in DNA, failed to increase editing efficiency or product purity compared with cells containing wild-type AAG. Indeed, even early generations of ABE recovered ≥99.9% pure product with a negligible rate of indels (≤0.1%) [13,75,88].

In its native context, TadA acts as a homodimer, with one monomer catalyzing deamination and the other monomer enabling tRNA substrate binding [89]. To optimize ABEs, Gaudelli et al. engineered a single-chain heterodimer comprised of a wild-type non-catalytic TadA monomer and evolved ecTadA monomer (TadA-TadA*). To improve editing efficiency, further optimization of ABE was performed. Extensive PACE and protein engineering resulted in seventh generation ABEs (ABE7.10), which converted target A-T to G-C efficiently (~50%) in human cells [13] (Table 1).

Only about one-quarter of pathogenic transition mutations encompass an appropriately located NGG PAM site that facilitates SpCas9-mediated base-editing. Unlike CBEs, which have proven to be broadly customizable with many Cas orthologs, ABEs have shown limited compatibility with Cas9 of any origin other than SpCas9. Although some homologs such as SaCas9 and circularly permuted Cas9 (CP-Cas9) have been adapted [88], editing efficiencies are substantially lower than those demonstrated with CBE counterparts [90]. This incompatibility is due to the low DNA-bound residence time of non-SpCas9, coupled with the slow enzymatic rate of deoxyadenosine deaminase. To address this problem, Richter et al. utilized phage-assisted continuous and non-continuous evolution (PACE and PANCE) methods to enhance the catalytic rate of the deoxyadenosine deaminase enzyme by 590-fold compared to that of ABE7.10 [77]. This next generation of ABEs, designated ABE8e, shows greatly enhanced activity and compatibility with diverse Cas9 homologs. As expected, the targeting scope of ABE8e also increased off-target RNA and DNA editing. However, the authors show that the off-target editing can be ameliorated by introduction of an additional point-mutation (V106W) [77]. Together, ABE8e expands the targeting range, editing efficiency and broad functionality of ABEs. It will be interesting to see whether these outcomes will translate in vivo and the eight-generation of ABEs can outperform previously developed base-editor constructs.

### 3.3. Prime-Editing

Despite the profound capabilities of CBEs and ABEs to edit the DNA, a major limitation of the current base-editing technologies (until recently) has been the ability to generate precise base-edits beyond the four transition mutations. Recently, a method to overcome these shortcomings, known as prime-editing, has been described by Anzalone et al. [10]. As with CRISPR-mediated base-editing, prime-editing does not rely on DSBs. Prime-editors use an engineered reverse transcriptase fused to Cas9 nickase and a prime-editing guide RNA (pegRNA) (Figure 1). Importantly, the pegRNA differs significantly from regular sgRNAs and plays a major role in the system’s function. The pegRNA contains not only (a) the sequence complimentary to the target sites that directs nCas9 to its target sequence, but also (b) an additional sequence spelling the desired sequence changes [10]. The 5′ of the pegRNA binds to the primer binding site (PBS) region on the DNA, exposing the non-complimentary strand. The unbound DNA of the PAM-containing strand is nicked by Cas9, creating a primer for the reverse transcriptase (RT) that is linked to nCas9. The nicked PAM-strand is then extended by the RT by using the interior of the pegRNA as a template, consequently modifying the target region in a programmable manner. The result of this step is two redundant PAM DNA flaps: the edited 3′ flap that was reverse transcribed from the pegRNA and the original, unedited 5′ flap. The choice of which flap hybridizes with the non-PAM containing DNA-strand is an equilibrium process, in which the perfectly complimentary 5′ would likely be thermodynamically favored. However, the 5′ flaps are preferentially degraded by cellular endonucleases that are ubiquitous during lagging-strand DNA synthesis [91]. Finally, the resulting heteroduplex containing the unedited strand and edited 3′ flap is resolved and stably integrated into the host genome via cellular replication and repair process.

The first generation of PEs (PE1) was comprised of Moloney murine leukemia virus reverse transcriptase (M-MLV RT), linked to the c-terminus of nCas9 and pegRNA, which was expressed on a second plasmid. The efficiency of PE1 reached maximum editing efficiency of 0.7–5.5% [10]. To further enhance the efficiency of the reverse transcriptase, Anzalone and colleagues tested different M-MLV RT variants that have been shown to enhance binding, enzyme processivity, and thermostability. Finally, as was previously applied to enhance editing in CBE and ABE systems, the authors directed a separate sgRNA to introduce a nick in the non-edited strand, thus directing DNA repair to that strand using the edited strand as a template. This yielded the latest generation prime-editor, designated PE3, which performed all 12 possible transition and transversion mutations (24 single-nucleotide substitutions) with average editing efficiencies of 33% (±7.9%) [10]. The number of off-target effects observed with PEs was greatly reduced, likely due to the need for complementation at Cas9 binding, PBS binding, and RT product complementation for flap resolution [10]. Prime-editing owns other advantages over previous CRISPR-mediated base-editing approaches, including less stringent PAM requirements due to the varied length of the RT template and no “bystander” editing. Notwithstanding, prime-editing is still in its infancy, and its specificity and potential for off-target modifications remains to be studied. The latest generations of base-editors are much closer characterized, particularly in vivo, and offer higher efficiency rates and lower-indel formation. Thus, at this stage these tools should be used over prime-editors whenever possible. In any case, the prime-editing system is an enormous milestone in the development of a universal method for genome editing, and its clinical adaptation towards the correction of known pathogenic mutations may prove tremendous.

## 4. Potential Applications of DNA Base-Editors and Prime-Editors

DNA base-editing and prime-editing has remarkable potential as a therapeutic tool to correct disease-causing mutations in the human genome. The ability to target nucleotides on either the plus or complementary minus DNA strand opens up the therapeutic applications of base-editors considerably. Over 25% of human pathogenic SNPs can be corrected by targeting the four transition mutations, and in principle prime-editing could correct up to 89% of the 75,122 pathogenic human genetic variants in ClinVar [10,46]. CRISPR-mediated HDR are confined to editing within dividing-cells, since these pathways are restricted to the S and G2 phases of the cell cycle. Conversely, base-editing employs cellular mismatch repair machinery and can be applied to reverse these defects in both dividing and terminally differentiated cell types [50]. Gene therapy is a major area where DNA base-editing and prime-editing toolkits can be applied because they have already been adapted to characterize, model, and correct the underlying causes of human genetic conditions. In the first generation of base-editors, Komor et al. converted *APOE4*, the most common genetic risk-factor in Alzheimer’s disease, into the lower-risk *APOE3r* variant in immortalized mouse astrocytes [46]. Additional studies have followed this initial example and demonstrated correction of pathogenic mutations in animal models [47,51,92,93,94], human cell lines [9,46,52,95], and even in human tripronuclear zygotes [96]. Below, we describe some of the advantages of DNA base-editing and prime-editing technologies for the treatment of inherited human diseases.

### 4.1. Editing Large Genes

DNA base-editing and prime-editing may prove to be particularly well adapted for correction of large genes (>4 kb). For many diseases, vector-mediated gene delivery is not feasible due to the cargo limit of viral vectors as well as the expression of multiple heterogeneous variants of the target gene [97,98]. Larger genes are relatively common and are involved in diseases such as cystic fibrosis, muscular dystrophy, and hemophilia A. The ABCA4 (coding sequence ~6.8 kb) and USH2A (coding sequence ~15.6kb) genes together account for almost 25% of all inherited retinal diseases [99]. Although some of the inherent limitations of viral vectors (and particularly AAVs) have been bypassed by elegant manipulation of the vector constructs [100], the utility of these systems is inherently limited by reduced transduction efficiency. Furthermore, even for genes that fit within the payload constrains of AAVs, regulating transgene expression can be challenging, and constitutive overexpression from strong or leaky promoters can result in undesirable outcomes such as cellular toxicity and off-target effects [101].

### 4.2. Targeting Autosomal Dominant Diseases

In autosomal recessive mutations, the loss of gene function can be compensated by introduction of a replacement allele into the cell. In contract, dominant negative mutations display not only impaired function, but also a novel phenotype that is pathogenic to the cell [102]. Thus, gene augmentation is not a suitable approach due to the requirement for silencing or ablating the defective gene in autosomal dominant diseases. Moreover, alternative RNA-based suppression and replacement using ribozymes, short hairpin RNAs, RNAi, and RNA editors-based approaches to silence or degrade both mutated and normal RNA transcripts are transient in nature and the limited durability of the therapy, regulation of gene expression levels, and target specificity have posed significant challenges for broad adaptation [103,104,105].

CRISPR-Cas mediated DNA base-editing and prime-editing presents an alternative approach to treat dominant diseases by addressing these challenges. For example, 25%–30% of the cases of retinitis pigmentosa (RP), the most common inherited retinal degeneration, are autosomal dominant [106]. Mutations in at least 23 genes have been reported to cause adRP to date, including over 180 mutations in the RHO gene, which accounts for over 25% of adRP cases. Approximately half of the RHO-associated adRP cases are caused by the P23H mutation [107]. Targeted adenine or cytosine-mediated based editing would convert P23H into P23R (CAC→CGC) or P23T (CAC→TAT/C), amino-acid substitutions that are probably well tolerated because they have not been reported to cause adRP.

### 4.3. Editing Premature Stop Codons

A large number of diseases including cystic fibrosis, Duchenne muscular dystrophy, β-thalassemia, and Usher syndrome are caused by premature stop mutations [108]. The disease phenotypes caused by premature stop mutations are frequently more severe than those that result from missense mutations, since premature stop mutations often result in a complete loss of protein function. Premature termination events are exacerbated by the spontaneous methylation of cytosine residues in context of CG (CpG), which create “hot spots” of C→T transitions that can generate the stop codon TAG upon epigenetic-mediated mutation of CGA [109]. Indeed, 12% of all mutations reported are single-point mutations that result in a premature termination codon (PTC) [110].

The therapeutic potential of DNA base-editors for correction of nonsense mutations has been demonstrated in vivo. Ryu and colleagues utilized an AAV targeted ABE to correct a premature stop codon (Q871X) in the *Dmd* gene [92]. Targeted base-editing restored dystrophin expression in 17% of myofibers, a level which is sufficient to improve muscle function. Lee et al. developed CRISPR-pass, a targeted tool for bypassing premature termination codons using CRISPR-mediated adenine base-editors that showed systematic rescue of all possible cases of PTCs [12]. This approach is applicable to 95.5% of clinically significant nonsense mutations in the ClinVar database. On the other hand, CRISPR-BEs can also be used to generate stop codons for gene KO studies or towards disruption of pathogenic genes [53]. Rossidis et al. injected AAV vectors encoding BE3 into embryonic mouse fetuses to disrupt the wild-type *Pcsk9* or *Hpd* gene through creation of a premature stop codon [54]. The authors were able to reduce plasma *Pcsk9* levels, a key regulatory protein controlling cholesterol levels and demonstrated rescue of the lethal hereditary tyrosinemia type 1 (HT1) phenotype.

### 4.4. Editing Splice-Site Variants

Alternative splicing is one of the most important post-transcriptional mechanisms, and it has been estimated that 15% of disease-causing point mutations affect pre-mRNA splicing [111]. Synthetic regulation of alternative splicing provides allows for selectively skipping mutation-containing exons while keeping normally functioning variants intact and presents a robust approach for targeting XL-RP and other inherited diseases. CRISPR-mediated CBEs and ABEs can disrupt donor/acceptor sites at AT/AA and GC/GG, respectively. Gapinske et al. demonstrated a novel method (CRISPR-SKIP) that utilizes cytidine deaminase single-base-editors to program exon skipping by mutating target DNA bases within splice acceptor sites [55]. The authors estimate that this approach could target ~63% (118,089 out of 187,636) of inner exons in protein coding transcripts. CRISPR-mediated modulation of RNA splicing was utilized to restore the frame of a mutant DMD gene and rescued its function in induced pluripotent stem cells (iPSCs) derived from a patient with Duchenne muscular dystrophy [112]. Recently, RNA-encoded ABE was delivered into the liver of Tyrosinemia I mice, correcting the splice-variant mutation and rescuing the phenotype [93].

### 4.5. Current Limitations

DNA base-editors and prime-editors, despite their relatively recent inception, have already been widely used in biomedical applications and hold great promise as a therapeutic strategy to address the underlying cause of debilitating human diseases. Base-editing and prime-editing technologies have grown and swiftly expanded in the past four years, with rapid advancements in their architecture to increase the underlying efficiency, targetability, and specificity. However, there remain many challenges to be overcome before the full potential of this platform can be realized. Base-editing technologies are still in their infancy, and further characterization of BEs and PEs in vivo is essential for enabling therapeutic applications. Much additional research is needed to further characterize and improve base-editing and prime-editing in a broad range of cell types and organisms. The off-target effects of Cas9 and BEs may differ, and a separate evaluation method is needed to better assess off target editing in a genome wide manner. Particularly, CBEs generate more indels, off-target editing, and undesired mutations than do ABEs [90], although we envision such constraints may be alleviated through additional cytidine deaminase engineering efforts. For instance, whole-genome sequencing of human iPSCs stably expressing an evolved CBE demonstrated C-to-T and C-to-G mutations outside the in silico predicted off-target sites. Importantly, the majority of the off-target mutations were C:G- > T:A transitions or C:G- > G:C transversions enriched for the APOBEC mutagenesis signature [113]. Compared with base-editing, prime-editing appears to be associated with lower off-target mutagenesis in human cell lines. Nevertheless, prime-editing could potentially introduce small insertions at the target site due to reverse-transcriptase mediated extension beyond the primer sequence. Moreover, while Anzalone and coworkers observed no differences in viability and minimum changes in the cellular transcriptome of cell lines expressing inactivated RT, the clinical viability and safety of in vivo prime-editing remain to be tested [10]. Finally, although Cas engineering has increased the scope of BEs and PEs, not all gene editing reagents are created equal, and additional efforts are necessary to broaden the range of targetable PAM sites. Indeed, many new variants are less efficient than the original BEs developed with nSpCas9. Further evolution of Cas9 proteins and discovery of new nucleases expanded PAM flexibility would broaden the scope of genome targeting while maintaining editing efficiency and specificity.

## 5. Delivery Systems

Safe and effective delivery of base-editors to target tissues or cells in the human body is one of the crucial and challenging factors for the therapeutic success not only of BEs and PEs but also of most Cas9-genome engineering reagents. Gene delivery systems (GDSs) can be classified into two categories based on the origin of the gene carrier. First, gene delivery systems may use non-viral delivery using physical (carrier-free gene delivery) and chemical approaches (synthetic vector-based gene delivery) (reviewed in [114,115]). Despite recent developments in non-viral delivery, therapeutic applications have been hindered by problems such as low transduction efficiency of target cells, cytotoxicity, and mutagenicity of the chromosomal DNA and have been reserved for in vitro and ex vivo delivery of CRISPR-Cas genome engineering regents to date.

The second category of GDS use recombinant viruses as gene carriers (reviewed in [116,117]). The use of viruses to delivery DNA encoding base-editors is a promising delivery modality and builds off the successes of gene therapy approaches. Several types of viruses, including retrovirus, lentivirus, adenovirus, adeno-associated virus, and herpes simplex virus have been modified in the laboratory for use in gene therapy applications [117]. Although these vector systems have unique strengths, in vivo application has been hampered by their respective limitations. Retroviral vectors can permanently integrate into the genome of infected cells but are prone to random integration and require mitotic cell division for transduction [118]. Adenoviral vectors possess a broad host range affinity of infectivity; however, immune elimination of infected cells often limits gene expression [119]. Herpes simplex virus can deliver large amounts of exogenous DNA; however, cytotoxicity and maintenance of transgene expression remain as obstacles [120]. Lentiviral vectors infect both dividing and non-dividing cells, but utility is restricted by concerns of off-target effects and potential oncogenesis [121]. Adeno-associated virus also targets many nondividing and dividing cell types but has a limited DNA packaging capacity [16,122]. Today, AAV vectors are the leading platform for in vivo delivery of gene therapies. There have been a number of excellent reviews concerning different delivery systems for CRISPR-based genome engineering [76,123]. Thus, here, we focus on the use of AAV vectors for delivery of the DNA base-editor and prime-editor toolkit and discuss the limitations and challenges of AAV-mediated delivery.

### Adeno-Associated Vectors

AAV is a single-stranded DNA parvovirus that belongs to the genus *Dependoparvovirus*. As indicated by its name and phylogenetic classification, AAV is a helper-dependent virus that requires the presence of another virus, including adenovirus and herpes simplex virus, to promote replication. The AAV genome is flanked by two inverted terminal repeats (ITRs), that contain *cis* elements required for replication and packaging [122] and the packaging-competent form of the AAV genome is ~4.7 kilobases (kb). AAV vectors have unique features that are beneficial for clinical applications and although ubiquitously prevalent in the human population, AAV has not been linked with any human disease and elicits mild immune response in humans. It rarely integrates into the host genome and can be preserved for extended periods of time in episomal forms, thus allowing for prolonged transgene expression. Since, AAVs are common in nature, many different serotypes exist, and as such display broad tissue-type and cell-type tropism profiles. Finally, the AAV genome is well characterized, so the consequences of genetic manipulations can reasonably be predicted [122]. For these reasons as well as others, AAVs have emerged as gold standards for in vivo gene delivery.

Importantly, the AAV ITRs, which induce transgene expression and play a central role in ensuring long-term cellular transduction, contain all the necessary *cis*-functions required for genome packaging. Thus, the basis for the production of recombinant AAV (rAAV) vectors is the fact that the *rep* and *cap* genes can be deleted from the viral genome and provided in *trans*. The viral genes can be replaced by a transgene with transcriptional control elements, resulting in a vector genome of approximately 4.5 kb flanked by the viral ITRs. The complete removal of viral coding sequences permits engineering of the AAV vector for gene therapy and contributes to their low cytotoxicity and immunogenicity as a delivery platform [122]. Indeed, substituting the *rep* and *cap* genes with an expression cassette containing a promoter (for example, human rhodopsin kinase (RK) promoter), a therapeutic transgene (*RPGR*, which encodes retinitis pigmentosa GTPase regulator), and polyadenylation signal forms the essence of AAV gene therapy vectors. To date, at least 12 natural serotypes and over 100 variants of AAV have been isolated and adapted as gene delivery vehicles with different tissue-type and cell-type tropism profiles. Researchers have pursued a variety of approaches to genetically engineer AAVs to enhance their transduction and production and to overcome immunity barriers in patients, including natural discovery, rational design, and directed evolution. While these efforts have played a substantial role in the advancement of these vectors for in vivo delivery, their discussion is beyond the scope of this article and are reviewed in depth elsewhere [124,125].

As of 13 May 2020, there are 107 ongoing interventional clinical trials involving rAAV registered at ClinicalTrials.gov. To date, there are three vectorized AAV serotypes that have gained regulatory approval for commercial use in patients: AAV1 (Glybera; uniQure (withdrawn in 2017), AAV2 (Luxturna; Spark Therapeutics/Novartis) and AAV9 (Zolgensma; AveXis/Novartis).

The advancement of AAV-base-editing and prime-editing technologies for in vivo translation faces the same set of challenges standing the development of AAV mediated therapeutic approaches in general, such as pre-existing anti-capsid immunity and vector-induced immunogenicity, therapeutic potency, persistence, and potential genotoxicity. The very recent report of deaths in a gene therapy trial sponsored by Audentes Therapeutics (acquired by Astellas in 2019) for children with X-linked myotubular myopathy is a tragic reminder of safety concerns surrounding AAV vectors. AAV-mediated delivery of CRISPR base-editing and prime-editing toolkits presents its own unique challenges. For example, the origin of CRISPR-Cas9 components in common bacteria raises the possibility of pre-existing humoral and cellular immunity, which may compromise safety or clinical efficacy [126]. Moreover, off-target editing is a critical concern that needs to be carefully assessed for any genome editing therapy. This concern may be exacerbated by the potential for long-term presence of rAAVs to result in genotoxicity through vector genome integration [127]. These topics are reviewed in-depth in a number of excellent reviews [122,128,129].

Another critical limitation of AAV vectors for delivery of base-editing and prime-editing reagents is their small carrying capacity compared with other viral vectors [98]. A CBE or ABE plus a guide RNA totals approximately 6 kbs, while, prime-editors are >7 kbs, well beyond the packaging constraints of AAV (Table 2). Several strategies have been investigated to maximize cargo capacity. Dual vectors consist of two independent vectors that hold a portion of the transgene cassette which is reconstituted following subsequent co-infection [130]. Examples of dual vector approaches to deliver genetic cargo beyond the packaging capacity of AAVs include *cis*-acting vectors in which regulatory elements are separated from the transgene of interest, *trans*-splicing vectors in which the viral cargo can be split into two or more rAAV vectors containing the adequately placed splice donor and acceptor sites, and overlapping vectors which take advantage of AAV’s ability to form concatemers via the homologous recombination of ITR sequences.

The use of these approaches has been successfully applied to deliver cytosine and adenine base-editors in vivo. Villiger et al. used an intein-mediated protein *trans*-splicing approach to deliver SaBE3 into the liver of adult mice harboring a point mutation in the *Pah* gene encoding phenylalanine hydroxylase [94]. Notably, the authors observed mRNA correction rates at frequencies of up to 63%, with no evidence of off-target base-editing. Similarly, Kim and co-workers utilized trans-splicing AAV to deliver split ABE7.10 into skeletal muscle in a mouse model of DMD [92]. Recently, Levy and colleagues report the application of *trans*-splicing inteins for delivery of split cytosine and adenine base-editors [131]. The optimized dual AAVs achieved durable in vivo base-editing in multiple tissues including the brain, liver, retina, heart, and skeletal muscles, with editing efficiencies of up to 59%.

Although dual vector approaches all increased the size of the cargo that can be packaged in AAV vectors and demonstrated in vivo proof of concept, these strategies, to date, are less efficient than single vector delivery, and have had variable success in animal models [130]. The choice of base-editor and dual vector delivery are important parameters. In addition, AAV capsid selection, dosage, and route of administration can have a profound impact on final editing efficiency [122]. Ongoing efforts, including in our lab, are focused on engineering AAV systems and optimizing base-editor systems for efficient vector-mediated delivery. The careful selection of both the CRISPR-base-editing construct and AAV platform will be essential to fully unleash the therapeutic potential of DNA base-editing and prime-editing tools.

## 6. Conclusions

DNA base-editing and prime-editing systems, with their simplicity and precision, hold great promise for the correction of point mutations in human genetic diseases. Two classes of base-editors described thus far have demonstrated proof of concept in generating precise point mutations in a wide variety of cell types and organisms. Recently, prime-editing has expanded the CRISPR-base-editing toolkit to all possible transition and transversion mutations. Research efforts to fine-tune BEs have greatly increased the efficiency, targeting scope, and purity of the edited product. Nevertheless, there remain many challenges to be overcome before the full potential of this platform can be realized. Perhaps the most pressing hurdle to overcome is the safe and effective delivery of base-editors and prime-editors to target tissues or cells in the human body. Still, the potential of DNA editing cannot be overstated, and it is evident that these tools will play a key role among the many CRISPR-derived tools to push forward the next frontier of personalized treatments.

## Figures and Tables

**Table 1:** Comparison of CRISPR-Base-editing Tools.

Base-Editor	Architecture	Editing Efficiency ^1^	Notes	Refs
BE1	rAPOBEC1-dCas9	0.8–7.7% in human cells	First-generation BE	[46]
BE2	rAPOBEC1-dCas9-UGI	Up to 20%	Prefers TC motifs	[46]
HF2-BE2	rAPOBEC1-HF2 nCas9-UGI	11.6–50%	Prefers TC motifs	[47,48]
BE3	rAPOBEC1-SpnCas9-UGI	Varies widely by cell type & target genes	Prefers TC motifs	[46,47,49,50,51,52,53,54,55,56,57,58,59,60,61,62]
HF-BE3	rAPOBEC1-HFnCas9-UGI	21 ± 3%	Reduced off-target editing	[52]
YE1-BE3	rAPOBEC1 (W90Y, R126E) SpnCas9-UGI	Comparable to BE3	Narrowed editing window	[63]
EE-BE3	rAPOBEC1 (R126E, R132E) SpnCas9-UGI	Comparable to BE3	Narrowed editing window	[63]
YEE-BE3	rAPOBEC1 (W90Y, R126E, R132E)-SpnCas9-UGI	Comparable to BE3	Narrowed editing window	[63]
VQR-BE3	rAPOBEC1-VQR SpnCas9-UGI	14.5–52%	Expanded PAM targeting	[63]
EQR-BE3	rAPOBEC1-EQR SpnCas9-UGI	7.5–35%	Expanded PAM targeting	[63]
VRER-BE3	rAPOBEC1-VRER SpnCas9-UGI	11–32%	Expanded PAM targeting	[63]
SaKKHBE3	rAPOBEC1-KKH SanCas9-UGI	14–62%	Expanded PAM targeting	[63]
FNLS-BE3	rAPOBEC1-SpnCas9-UGI	41–93%	Additional N-terminus NLS; Increased editing efficiency	[62]
RA-BE3	rAPOBEC1 (RA)-SpnCas9-UGI	30–58%	Increased editing efficiency	[62]
A3A-BE3	hAPOBEC3A-SpnCas9-UGI	22.5%	Preferential deamination of cytidines in a TCR motif	[59]
eA3A-HF1-BE3-2xUGI	APOBEC3A-HF1 SpnCas9-UGI-UGI	17.5%	Deaminates cytosines with preference TCR > TCY > VCN; Increased editing efficiency	[59]
eA3A-Hypa-BE3-2xUGI	APOBEC3A-Hypa SpnCas9-UGI-UGI	14%	Deaminates cytosines with preference TCR > TCY > VCN; Increased editing efficiency	[59]
hA3A-BE3	hAPOBEC3A-SpnCas9-UGI	2–62%	Efficient editing in methylated region and in GpC context	[64]
hA3B-BE3	hAPOBEC3B-SpnCas9-UGI	2–62%	Intermediate editing efficiency	[64]
hA3G-BE3	hAPOBEC3G-SpnCas9-UGI	2–62%	Greatly decreased editing efficiency	[64]
hAID-BE3	hAPOBEC3A-SpnCas9-UGI	2–62%	Intermediate editing efficiency	[64]
SaCas9-BE3	rAPOBEC1-SanCas9-UGI	∼50–75%	Expanded targeting range	[63]
xCas9-BE3	rAPOBEC1-xnCas9-UGI	37% (NGG PAM)	Expanded targeting range	[60]
ScCas9-BE3	rAPOBEC1-ScnCas9-UGI	19–41%	Affinity to minimal 5′-NNG-3′ PAM sequences	[65]
SniperCas9-BE3	rAPOBEC1-SnipernCas9-UGI	0.04–50%	Increased sgRNA scope; further reduced off-target activities	[33]
iSpyMac-BE3	rAPOBEC1-iSpyMacnCas9-UGI	50%	Elevated editing efficiencies on 5′-NAAN-3′ targets	[66]
Target-AID	SpnCas9-CDA1-UGI	17–55%	First-generation base-editor	[8]
Target-AID-NG	SpnCas9 (NG)-CDA1-UGI	1–38%	Expanded targeting range	[67,68]
CRISPR-X	SpdCas9-MS2-hAID	N/A	High activity; used for random mutagenesis	[69]
TAM	SpdCas9-hAID (P182X)	N/A	High activity; used for random mutagenesis	[70]
BE-PLUS	SunTag-SpnCas9-scFv-rAPOBEC1-UGI	2–38%	Broadened targeting window; reduced off-target editing	[71]
BE4	rAPOBEC1-SpnCas9-UGI-UGI	Varies widely by cell type & target genes	Increased editing efficiency	[47,49,61,72]
BE4-Gam	Gam-rAPOBEC1-SpnCas9-UGI-UGI	17–58%	Increased editing efficiency and product purity	[49,61]
BE4-Max	rAPOBEC1-SpnCas9-UGI-UGI	69–77%	Codon optimized for mammalian cells	[72]
AncBE4-Max	rAPOBEC1-SpnCas9-UGI-UGI	75–84%	Ancestral reconstruction of the deaminase component	[72]
SaCas9-BE4	rAPOBEC1-SanCas9-UGI-UGI	25–60%	Expanded PAM targeting	[49]
SaCas9-BE4-Gam	Gam-rAPOBEC1-SanCas9-UGI-UGI	42–67%	Increased editing efficiency and product purity	[49]
evoBE4max	rAPOBEC1-SpnCas9-UGI-UGI	Up to plateau levels (~60–80%)	Improved efficiency in GC context	[73]
evoFERNY-BE4max	rAPOBEC1-SpnCas9-UGI-UGI	Up to plateau levels (~60–80%)	29% smaller than APOBEC1	[73]
Cas12a-BE	rAPOBEC1-dLbCpf1-UGI	3–46%	Can target T-rich PAM sequence	[74]
ABE7.8/9/10	ecTadA-ecTadA *-SpnCas9	1.7–20%	First generation ABE	[13]
xCas9-ABE7.10	ecTadA-ecTadA *-nxCas9	69% (NGG PAM)	Expanded PAM targeting	[60]
VQR-ABE	ecTadA-ecTadA *-Sp VQR nCas9	20%	Expanded PAM targeting	[75]
Sa(KKH)-ABE	ecTadA-ecTadA *-Sa KKH nCas9	16%	Expanded PAM targeting	[75]
ABEmax	ecTadA-ecTadA *-SpnCas9	27–52%	Improved editing efficiency	[72]
ABE7.10max	ecTadA-ecTadA *-SpnCas9	19.2–40.7%	Improved editing efficiency	[76]
ABE8e	ecTadA-ecTadA *-SpnCas9	18%–86%	Improved editing efficiency	[77]
PE1	dSpCas9-MMLV-RT	0.7–5.5%	First generation PE	[10]
PE2	dSpCas9-MMLV-RT	1.6- to 5.1-fold improvement over PE1	Targets all transition/transversion mutations; small indels	[10]
PE3	nSpCas9-MMLV-RT	20–50%	Targets all transition/transversion mutations; small indels	[10]

^1^ Editing efficiency in vitro unless otherwise stated. * Engineered TadA domain.

**Table 2:** Genetic Payload of Base-editing Tools.

CRISPR-Tool	Function	Gene Size (kb)
SpCas9	Nuclease	4.2
SaCas9	Nuclease	3.2
CjCas9	Nuclease	2.9
xCas9	Nuclease	3.7
AsCpf1	Nuclease	3.9
LbCpf1	Nuclease	3.7
rAPOBEC1	Cytosine Deaminase	0.7
ecTadA(8e)-dimer	Adenine Deaminase	1.2
MMLV RT	Reverse Transcriptase	2.2
UGI	Inhibits UNG	0.3
BE4	Cytosine Base-editor	5.6
ABE7.10	Adenine Base-editor	5.3
PE2	Prime Editor	6.4

## Abbreviations

AAG	Alkyl adenine DNA glycosylase
AAP	Assembly activating protein
AAV	Adeno-associated virus
ABE1.2	First-generation adenine deaminase
ABE7.10	Seventh-generation adenine base-editor
ABE8	Eighth-generation adenine base-editor
AID	Activation-induced cytidine deaminase
APOBEC1	Apolipoprotein B MRNA Editing Enzyme Catalytic Subunit 1
AsCpf1	*Acidaminococcus* sp. Cfp1
LbCpf1	*Lachnospiraceae bacteriumare* Cpf1
Bps	Base-pairs
SaCas9	Staphylococcus aureus Cas9
BE	Base-editor
BER	Base excision repair
Cas	CRISPR-associated genes
CBE1	First-generation cytosine base-editor
CBE2	Second-generation cytosine base-editor
CBE4	Fourth-generation cytosine base-editor
CjCas9	*Campylobacter jejuni* Cas9
Cpf1/Cas12a	Prevotella and Francisella1
CRISPR	Clustered Regularly Interspaced Short Palindromic Repeats
dAsCpf1	Catalytically inactive AsCpf1
dCas9	Catalytically deficient Cas9
dLbCpf1	Catalytically inactive LbCpf1
DSB	Double-strand DNA break
CBE	Cytosine base-editor
ABE	Adenine base-editor
PE	Prime editor
Indel	Insertion or deletion
NHEJ	Non-homologous end joining
GDS	Gene delivery system
HDR	Homology directed repair
HEK293	Human embryonic kidney cells
HF	High-fidelity version of dCas9
HT1	Hereditary tyrosinemia type 1
iPSCs	Induced pluripotent stem cells
ITR	Inverted terminal repeats
M-MLV RT	Moloney murine leukemia virus reverse transcriptase
nCas9	Cas9 nickase
NLS	Nuclear Localization Signal
ORF	Open reading frame
PACE	Phase assisted continuous evolution
PAM	Protospacer adjacent motif
gRNA	Guide RNA
PANCE	Phage-assisted non-continuous evolution
PBS	Primer binding site
PE1	First-generation prime editor
pegRNA	Prime editing guide RNA
pmCDA1	Petromyzon marinus cytidine deaminase 1
rAAV	Recombinant AAV
RK	Rhodopsin kinase
RP	Retinitis pigmentosa
RT	Reverse Transcriptase
ScCas9	*Streptococcus canis* Cas9
sgRNA	Small guide RNA
SpCas9	S. pyogenes’ Cas9
Spy-mac	Cas9 fusion derived from Streptococcus pyogenes and Streptococcus macacae
TadA	tRNA adenosine deaminase
TALEN	Transcription activator-like effector nucleases
tracrRNA	Trans-activating RNA
UNG	Uracil N-glycosylate
ZFN	Zinc-finger nuclease
